# Effects of dietary Na^+ ^deprivation on epithelial Na^+ ^channel (ENaC), BDNF, and TrkB mRNA expression in the rat tongue

**DOI:** 10.1186/1471-2202-10-19

**Published:** 2009-03-12

**Authors:** Tao Huang, Frauke Stähler

**Affiliations:** 1 German Institute of Human Nutrition Potsdam-Rehbruecke, Department of Molecular Genetics, Arthur-Scheunert-Allee 114-116, 14558 Nuthetal, Germany

## Abstract

**Background:**

In rodents, dietary Na^+ ^deprivation reduces gustatory responses of primary taste fibers and central taste neurons to lingual Na^+ ^stimulation. However, in the rat taste bud cells Na^+ ^deprivation increases the number of amiloride sensitive epithelial Na^+ ^channels (ENaC), which are considered as the "receptor" of the Na^+ ^component of salt taste. To explore the mechanisms, the expression of the three ENaC subunits (α, β and γ) in taste buds were observed from rats fed with diets containing either 0.03% (Na^+ ^deprivation) or 1% (control) NaCl for 15 days, by using *in situ *hybridization and real-time quantitative RT-PCR (qRT-PCR). Since BDNF/TrkB signaling is involved in the neural innervation of taste buds, the effects of Na^+ ^deprivation on BDNF and its receptor TrkB expression in the rat taste buds were also examined.

**Results:**

*In situ *hybridization analysis showed that all three ENaC subunit mRNAs were found in the rat fungiform taste buds and lingual epithelia, but in the vallate and foliate taste buds, only α ENaC mRNA was easily detected, while β and γ ENaC mRNAs were much less than those in the fungiform taste buds. Between control and low Na^+ ^fed animals, the numbers of taste bud cells expressing α, β and γ ENaC subunits were not significantly different in the fungiform, vallate and foliate taste buds, respectively. Similarly, qRT-PCR also indicated that Na^+ ^deprivation had no effect on any ENaC subunit expression in the three types of taste buds. However, Na^+ ^deprivation reduced BDNF mRNA expression by 50% in the fungiform taste buds, but not in the vallate and foliate taste buds. The expression of TrkB was not different between control and Na^+ ^deprived rats, irrespective of the taste papillae type.

**Conclusion:**

The findings demonstrate that dietary Na^+ ^deprivation does not change ENaC mRNA expression in rat taste buds, but reduces BDNF mRNA expression in the fungiform taste buds. Given the roles of BDNF in survival of cells and target innervation, our results suggest that dietary Na^+ ^deprivation might lead to a loss of gustatory innervation in the mouse fungiform taste buds.

## Background

In rodents, salt taste is mainly processed by the taste buds in the fungiform papillae spread across the anterior tongue, where the taste bud cells are innervated by chorda tympani (CT) nerves [1-3]. Previous studies have shown that sodium deprivation leads to a reduction in taste neuron responses of the CT nerves to lingual NaCl stimulation, while the responses to other taste stimuli remain unchanged [4,5]. In the nucleus of the solitary tract (NST) and the parabrachial nucleus (PBN), the first and the second relays of central taste system, dietary Na^+ ^deprivation also reduces nerve responses to lingual NaCl stimulation [6-8]. Taken together, this indicates that the taste responses to NaCl from CT nerves, and NST and PBN taste neurons are consistently reduced following dietary Na^+ ^deprivation, which suggests that the Na^+ ^deprivation might regulate salt taste perception and/or transduction at the peripheral taste system.

In the rat tongue, the amiloride sensitive epithelial Na^+ ^channel (ENaC) is considered as the "receptor" element for the Na^+ ^component of salt taste [1,2,9-12]. Prior whole-cell recording experiments showed that in the anterior tongue, which is sensitive to salt taste, amiloride sensitive currents are observed in two thirds of fungiform taste receptor cells (TRCs), whereas in the posterior tongue, none of vallate TRCs is amiloride sensitive [1,2]. During dietary Na^+ ^deprivation, however, the number of amiloride sensitive TRC and the current amplitude increase in the fungiform taste buds, and an amiloride sensitive current is induced in about half of the vallate taste buds cells [13]. Furthermore, Na^+ ^deprivation increases apical accumulation of ENaC subunits in the taste bud cells. The differential expression and localization of ENaC might lead to an up regulation of ENaC function following Na^+ ^deprivation [13].

Altogether, dietary Na^+ ^deprivation increases the function of ENaC expressed in TRCs, while it decreases the responses of CT nerves as well as NST and PBN taste neurons to NaCl solution. The underlying mechanisms are still unclear.

In peripheral sensory system, experience- or activity-dependent rearrangement of nerve innervation is a general property. For example, deprivation of smell reduces the afferent neural innervation in the rat olfactory bulb. During the process, BDNF expression is concomitantly downregulated [14]. In contrast, visual experience upregulates BDNF expression in the rat retina [15]. Both studies suggest a positive correlation between experience and BDNF level and open the possibility for a similar mechanism in the taste system. In the taste system, the expression of BDNF in taste buds is a key factor in appropriate gustatory innervation [16]. One recent transgenic experiment showed that BDNF expression in the anterior tongue functioned as a chemoattractant that allowed CT fibers to distinguish their fungiform papilla targets from non-gustatory epithelium such as filiform papillae [17]. Since taste bud cells regenerate approximately every ten days, the BDNF guided innervations must be constantly reformed. During a low Na^+ ^feeding, the decrease of CT nerve response to lingual Na^+ ^stimulation, could be a consequence of interrupted innervations of taste bud cells due to a reduction in BDNF expression.

To explore the gustatory mechanisms of dietary Na^+ ^deprivation we examined ENaC subunit mRNA expression in the taste buds of rats fed with a diet containing either 0.03% NaCl (Na^+ ^deprivation) or 1% NaCl (control), using *in situ *hybridization and real-time quantitative RT-PCR (qRT-PCR). Further the mRNA expression of BDNF and its receptor TrkB in the taste buds were compared between control and Na^+ ^deprived animals.

## Results

### Effect of Na^+ ^deprivation on ENaC

**Figure 1:**
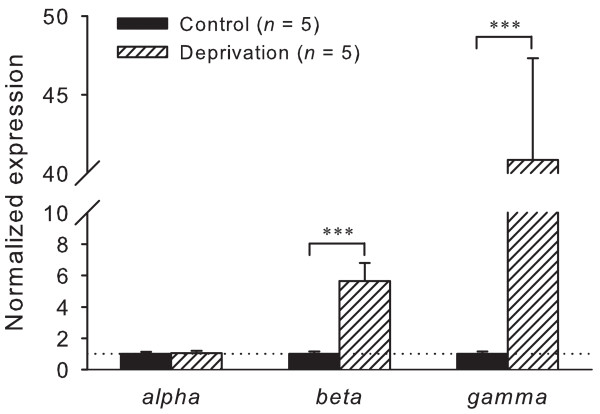
**Normalized expression of α, β and γ ENaC mRNA in the rat distal colon**. Expression levels were normalized to each sample's respective GAPDH expression and then reported as a fold of control (mean ± S.E.M). The data of control were from rats fed with a diet containing 1% NaCl, and the data of the deprivation were from those fed with a diet containing 0.03% NaCl for 15 days. *n *indicates the number of animals used. Next figures are the same. *** *p *< 0.001.

To identify Na^+ ^deprivation condition, 24-h urinary Na^+ ^excretions of control and 15-day Na^+ ^deprived rats were measured by flame photometry. Rats fed low Na^+ ^diet excreted 0.054 ± 0.014 mmol Na^+^. Rats on the 1% NaCl diet excreted 1.79 ± 0.19 mmol Na^+^. These results are consistent with previous Na^+ ^deprivation studies [5,18]. Therefore, it served as positive control for our methodology. Furthermore, we compared α, β and γ ENaC mRNA expression in the distal colon between control and Na^+ ^deprived animals because previous studies have clearly shown that low Na^+ ^diet feeding dramatically increased β and γ ENaC mRNA expression in the rat distal colon, while α ENaC did not change at all [19,20]. Figure 1 shows quantitative ENaC mRNA expression of control and Na^+ ^deprived animals. Randomization analyses revealed that dietary Na^+ ^deprivation had no effect on α ENaC subunit expression in the distal colon (*p *> 0.05), whereas the deprivation upregulated β and γ ENaC mRNA levels up to 6 and 47 folds, respectively. These findings supported previous report [6], approving the functionality of our experimental set up.

### Effect of Na^+ ^deprivation on ENaC mRNA expression in the tongue

**Figure 2:**
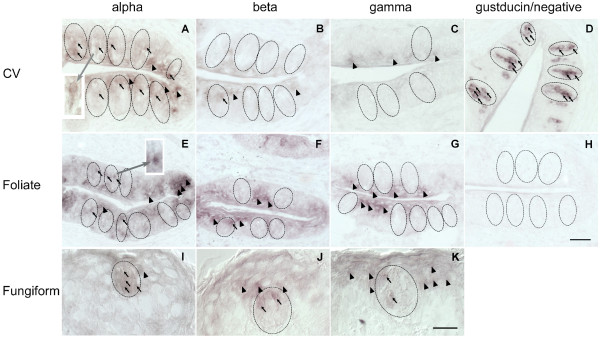
***In situ *hybridization analysis of α, β and γ ENaC subunits in vallate (A-C), foliate (E-G) and fungiform (I-K) papillae of control rats**. In each row, the sequence is α, β, γ from left to right. D and H are positive (α gustducin mRNA) and negative (using sense probe) controls on circumvallate papillae, respectively. Outlines of taste buds are shown by dotted lines. Arrows indicate stained cells within taste buds and arrowheads indicate those outside of taste buds. Higher magnification of α ENaC subunit in vallate and foliate taste buds is indicated in A and E, respectively. Scale bars are 50 μm for A-H and 25 μm for I-K.

*In situ *hybridization was used to localize ENaC mRNA in the rat tongue. In both control and Na^+ ^deprived animals, α, β and γ ENaC mRNAs were detected in fungiform taste bud cells, where α ENaC mRNA was more widely distributed compared to β, and γ (Fig. 2I–K). However, in vallate and foliate taste bud cells only signals for the α ENaC subunit mRNA were easily found, while those for the β and γ subunits were almost absent in both groups of animals. In both anterior and posterior parts of the tongues, all three ENaC subunits could be easily found in non-chemosensory lingual epithelia as well (Fig. 2).

To compare ENaC subunit expression in fungiform, vallate and foliate taste buds between control and Na^+ ^deprived animals, the expression ratios were analyzed based on positively stained cells across total taste bud cells per section (table 1). Gustducin served as control, since it is expressed in TRC as a downstream molecule of Tas2rs and Tas1rs [21]. Although β and γ ENaC-positive cells were nearly absent in control and Na^+ ^deprived animals, their expression ratios were comparable. One-way ANOVA indicated that in fungiform, vallate and foliate taste buds, the ratios of α, β and γ ENaC- and gustducin-positive cells were not significantly different between control and Na^+ ^deprived rats (*p *> 0.05).

**Table 1:** ENaC- and gustducin-positive cell across total taste bud cells per sections

	**Fungiform papilla**		**Vallate papilla**		**Foliate papilla**	
			
ratio ± SEM	control	Na^+ ^deprived	control	Na^+ ^deprived	control	Na^+ ^deprived
	*n *= 3	*n *= 3	*n *= 3	*n *= 3	*n *= 3	*n *= 3
						
α ENaC	0.49 ± 0.040	0.53 ± 0.035	0.18 ± 0.016	0.15 ± 0.019	0.31 ± 0.026	0.27 ± 0.024
β ENaC	0.15 ± 0.015	0.17 ± 0.012	0.02 ± 0.007	0.03 ± 0.003	0.03 ± 0.004	0.03 ± 0.005
γ ENaC	0.15 ± 0.011	0.14 ± 0.016	0.01 ± 0.004	0.02 ± 0.003	0.01 ± 0.006	0.02 ± 0.005
gustducin	0.13 ± 0.017	0.11 ± 0.010	0.29 ± 0.034	0.26 ± 0.032	0.35 ± 0.046	0.37 ± 0.035

*n *indicates the number of animals used in the experiments.

**Figure 3:**
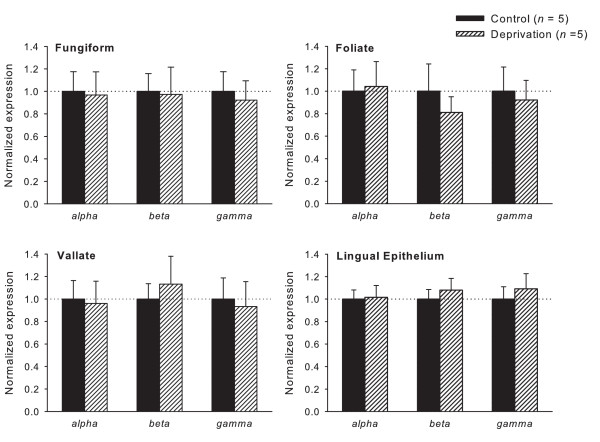
**Normalized expression of α, β and γ ENaC mRNA in rat fungiform, foliate and vallate taste buds, and non-chemosensory epithelium**. Expression levels were normalized to each sample's respective GAPDH expression and reported as a fold of control. In sodium deprived rats, the expression levels of ENaC subunits from fungiform, foliate and vallate taste buds, as well as lingual epithelium, were not different from those in control animals. *n *indicates the number of animals used in the experiments.

To confirm the data of the *in situ *hybridization analyses, real-time qRT-PCR was performed using taste bud cDNA from control and Na^+ ^deprived rats. The ENaC mRNA expression levels of the two groups for fungiform, vallate and foliate taste buds, and non-chemosensory lingual epithelia were compared. Randomization analyses performed by "Relative Expression Software Tool" (REST) indicated that all three ENaC subunit mRNA levels in taste buds and non-chemosensory lingual epithelia were equal between control and Na^+ ^deprived animals (Fig. 3). These results were consistent with the *in situ *hybridization analysis. Thus, dietary Na^+ ^deprivation had no effect on any ENaC subunit mRNA expression in the rat tongue.

### Effect of Na^+ ^deprivation on BDNF and TrkB mRNA expression in taste buds

**Figure 4:**
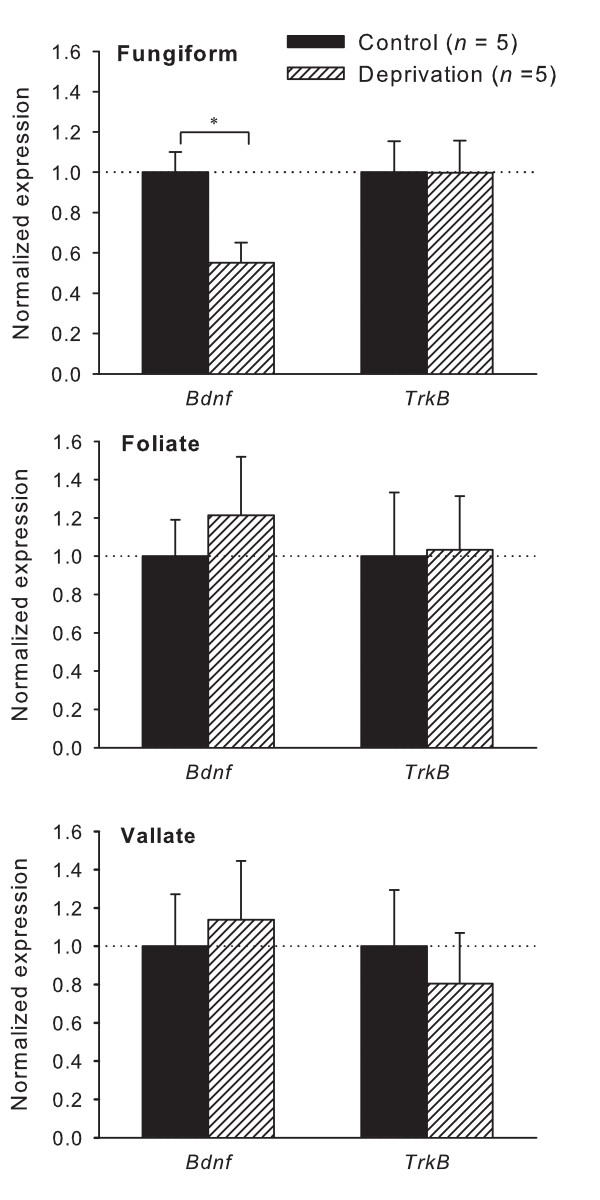
**Normalized expression of BDNF and TrkB mRNA in the rat fungiform, vallate and foliate taste buds**. Expression levels were normalized to each sample's respective GAPDH expression and reported as a fold of control. In sodium deprived rats, BDNF mRNA expression in fungiform taste buds was significantly downregulated. *n *indicates the number of animals used in the experiments. * *p *< 0.05.

In order to explore if dietary Na^+ ^deprivation has any effect on peripheral taste innervations, the mRNA expression of BDNF and its specific receptor TrkB was analyzed in rat taste buds. Expression profiling via real-time qRT-PCR revealed a significant downregulation of BDNF mRNA up to 50% (*p *= 0.017) in fungiform taste buds of Na^+ ^deprived animals, while TrkB mRNA expression levels did not change (Fig. 4). In vallate and foliate taste buds, both BDNF and TrkB mRNA expression levels were not different between control and experimental groups (Fig. 4). Taken together, dietary Na^+ ^deprivation specifically reduced BDNF mRNA expression in the rat fungiform taste buds.

## Discussion

Our in *situ *hybridization analysis demonstrated that all three ENaC subunits are expressed in the rat fungiform taste buds. The α ENaC subunit is expressed more extensively as compared to the β and γ ENaC subunits. On the other hand, in vallate and foliate taste buds, only α ENaC subunit is easily found, while β and γ ENaC subunits are nearly absent. The results are in line with those observed in rats [10], mice [22] and human [23]. Using immunohistochemical analysis, Lin *et al*. have demonstrated similar expression pattern of α, β and γ ENaC protein in the rat taste buds. They also found that α ENaC protein is expressed in large amounts in the fungiform, vallate and foliate taste buds, whereas β and γ subunits are expressed in minimal amounts in vallate and foliate taste buds [10,13,24]. Thus, there is an excellent agreement between ENaC mRNA expression observed here and protein distributions in the rat taste buds, suggesting that ENaC subunit expression in rat taste buds mainly depends upon the mRNA expression.

Dietary Na^+ ^deprivation increases ENaC subunit protein expression in both fungiform and vallate papillae [13]. In the present study, however, ENaC subunit mRNA expression is not altered in fungiform, foliate and vallate taste buds after dietary Na^+ ^deprivation. This suggests that Na^+ ^deprivation modulates functional ENaC expression in a posttranscriptional way. Serum- and glucocorticoid-inducible kinase (SGK), an aldosterone induced kinase, and ubiquitin-protein ligase Nedd4-2 might be involved in this process, since previous studies have shown that the interaction between SGK and Nedd4-2 controls ENaC expression at the cell surface via protein-trafficking mechanisms [25-27]. Given the fact that dietary Na^+ ^deprivation increases plasma aldosterone, this could increase the abundance of ENaC in the plasma membrane through the SGK/Nedd4-2 pathway in Na^+ ^deprived rats, while ENaC mRNA expression remains unchanged.

In the present study, the decrease of BDNF mRNA in fungiform taste buds is tied to an environmental factor such as dietary Na^+ ^deprivation. This indicates that the expression of BDNF in the taste buds is regulated by the integrity of sensory input, such as constant ingestion of NaCl. In olfactory and visual systems, BDNF expression is also regulated by olfactory and visual stimulation [14,15,28-30]. Thus, it seems that this mechanism is a popular phenomenon in sensory systems.

The specific receptor of BDNF, TrkB, is also involved in gustatory innervations. For instance, in TrkB knockout mice there is no gustatory innervation of taste buds [31]. This suggests that TrkB, expressed on the axons of the primary neurons, mediates BDNF guided innervations [32,33]. However, there is no consistent alteration in TrkB expression during dietary Na^+ ^deprivation. These results are similar with those observed in the visual cortex, where light deprivation results in decreased BDNF mRNA without a concomitant change in TrkB mRNA [28]. Moreover, *in vitro *culture experiments showed that TrkB expression is independent of BDNF expression [34]. Therefore, TrkB expression in taste buds seems to be independent of BDNF availability.

Dietary Na^+ ^deprivation reduces BDNF mRNA expression in fungiform taste buds, but does not affect its expression in foliate and vallate taste buds. As amiloride sensitive salt taste is mainly processed through fungiform taste bud cells [1,9-11], the downregulation of BDNF might be the consequence of reduced ENaC-mediated Na^+ ^influx into taste bud cells following Na^+ ^deprivation. Prior electrophysiological experiments have shown that functional ENaC localizes in Na^+^-chemosensitive cells within rat fungiform papillae. These cells are speculated to communicate with presynaptic (type III) taste bud cells, which then can relay salt taste information to nerve endings via conventional synaptic transmission [12]. Although *in vitro *experiments demonstrate that dietary Na^+ ^deprivation increases the function of ENaC in fungiform papillae [13], the low-salt diet would reduce the stimulation of lingual ENaC *in vivo*, which in turn could decrease neurotransmitter activity in the ENaC expressing taste receptor cells and the related presynaptic cells with subsequent decrease in intracellular signaling pathways. It is now widely recognized that neuronal activity regulates BDNF expression (for review, see [35]). The decreased neuronal activities might reduce BDNF expression in the ENaC expressing cells and the presynaptic cells because BDNF is present in receptor cells and presynaptic cells in the fungiform papillae [36]. On the other hand, the receptor of BDNF, TrkB, is also found in presynaptic cells, and in intragemmal nerve endings [36]. Given the roles of BDNF in survival of cells and target innervation, it might exert some effects on gustatory innervation and have an autocrine and/or paracrine effect on taste bud cells [37,38]. The long-term (15 days) decrease of BDNF would lead to the related presynaptic cell death and the loss of BDNF-sustained gustatory innervation. The occurrence of these events could be salt taste specific because the peripheral taste system maintains a specific relationship between taste bud cells and geniculate ganglion cells [39-41]. However, other interpretations are possible because we cannot prove a specific loss of innervation after dietary Na^+ ^deprivation.

In this study, long-term Na^+ ^deprivation reduces BDNF expression in the related receptor and presynaptic taste cells, which in turn could possibly result in a loss of gustatory innervation. This might be the reason why dietary Na^+ ^deprivation leads to a specific reduction in salt taste responses in the peripheral and central gustatory system. However, such mechanisms are probably not the only ones involved in taste-evoked responses to NaCl. Dietary Na^+ ^deprivation elicits aldosterone secretion, which can enhance ENaC function in taste receptor cells and increase salt intake in rats. We hypothesize that at early stage of Na^+ ^deficiency, aldosterone may play a predominant role in the neural responses to lingual NaCl stimulation, which is confirmed by acute Na^+ ^depletion experiments in the NST and the PBN [42,43]. As mentioned above, prolonged Na^+ ^deprivation could result in a loss of gustatory innervation, which would reduce the neural responses to NaCl stimulation, but the role of aldosterone in ENaC function and salt intake may remain unchanged.

## Conclusion

The current study shows that 15-day dietary Na^+ ^deprivation has no effect on α, β and γ ENaC mRNA expression in the rat fungiform, vallate and foliate taste buds, and non-chemosensory lingual epithelium. Combining with previous findings that dietary Na^+ ^deprivation enhances ENaC protein expression at the apical site in the rat taste buds, our findings suggest that dietary Na^+ ^deprivation regulates ENaC expression in a posttranscriptional way. On the other hand, Na^+ ^deprivation specially downregulates BDNF expression in fungiform taste buds but not in vallate and foliate taste buds, which leads to the hypothesis that dietary Na^+ ^deprivation might reduce the neural innervation of the fungiform taste buds.

## Methods

### Animals

All experimental protocols received prior approval from the Ministry of rural development, environmental and consumer protection of Germany, and animal experiments were carried out in accordance with the National Institute of Health Guide for the Care and Use of Laboratory Animals.

Experiments were performed using adult (6–8 weeks old) male Sprague-Dawley rats weighing 240–300 g (on experiment day). Control animals were fed with a diet containing 1% NaCl, and experimental animals were fed with a diet containing 0.03% NaCl for 15 days. Both diets were purchased from Ssniff (Soest, Germany). They were identical except for NaCl concentration. The animals were supplied with food and deionized water *ad libitum*. On the experiment day, the animals were anesthetized by CO_2 _narcosis, and killed by cervical dislocation. Tongues and distal colons were rapidly dissected. For *in situ *hybridization experiments, tongues were immediately frozen in liquid nitrogen, and then stored at -80°C. For RNA extraction, distal colons were first flushed with isotonic saline, and then stored in RNAlater (Ambion, Germany) at -20°C for later RNA isolation.

### Isolation of taste buds

Fungiform, foliate, and vallate papillae were isolated according to previously published procedures with a few changes [10,24,44]. Briefly, the freshly isolated tongue was immediately immersed in cold Tyrode solution (140 mM NaCl, 5 mM KCl, 1 mM CaCl_2_, 1 mM MgCl_2_, 10 mM Hepes, 5 mM glucose, 5 mM pyruvat-Na, pH 7.4) for 10 min. Lingual epithelium was separated from connective tissue by enzymatic dissociation (1 mg collagenase A and trypsin inhibitor, and 0.5 mg dispase-I per ml in Tyrode solution). The peeled epithelium was pinned down with the inner side upwards in a Petri dish, and incubated in Ca^2+ ^free Tyrode solution: [140 mM NaCl; 2 mM KCl; 10 mM Hepes; 2 mM BAPTA (1,2-Bis (2-aminophenoxy) ethane-N, N, N, N-tetraacetic acid tetrapotassium salt), pH 7.4] for 25–40 min. Taste buds were isolated under a binocular microscope. For fungiform papillae, taste buds were individually suctioned by using a polished pipette with 50 μm tip. For foliate or vallate papilla, a pipette with a 100 μm opening was used to collect all taste buds. In addition, the epithelium of the very center of the tongue, known as "taste blind" area, was collected to serve as non-chemosensory control tissue. The non-chemosensory lingual epithelia and the taste buds from three papillae were each transferred into separate tubes containing RNAlater. All samples were stored at -80°C until RNA extraction.

### RNA and cDNA preparation

#### Lingual tissue

Total RNA from tongue epithelium and taste buds were extracted using RNeasy mini-columns (QIAGEN, Hilden, Germany). DNase I (included in the kit) treatment was applied to eliminate traces of DNA during the procedure. After the extraction, the quality and quantity was analyzed with Bioanalyzer 2100 (Agilent, Santa Clara, CA, USA). Reverse transcription was performed using 400 U Superscript II RNase H- Reverse Transcriptase (Invitrogen, Germany) and 3 μg random primers (Invitrogen) in 20-μl reaction volume containing 1 × First strand buffer (Invitrogen), 0.5 mM dNTPs, and 20 U of RNase inhibitor. After incubation for 60 minutes at 42°C, the reaction was stopped by heating (15 minutes at 72°C). To check if there was genomic DNA contamination, RNA was also treated in parallel in the absence of reverse transcriptase, and the material was then used for PCR.

#### Distal Colon

TRIzol Reagent (Invitrogen) was used to isolate total RNA from distal colon. First, 50–100 mg tissue was homogenized in 1 ml of TRIzol reagent, and RNA was extracted with phenol-chloroform. After precipitation with isopropanol, the RNA was rinsed with 75% ethanol and resuspended in sterile water. Then RNA was quantified by optical density and the integrity was checked on a 1% agarose gel. cDNA was synthesized from 1.0 μg of total RNA, which was treated with DNase I (1 U, Invitrogen) for 60 min. The other steps were similar with that of the lingual tissue.

### *In situ *hybridization analysis

#### cRNA probe preparation

All three ENaC cDNAs were cloned in order to synthesize RNA probes. The cDNAs were obtained from rat kidney total RNA by reverse transcription coupled to PCR. The primers used were as follows.

α ENaC, 5'-CCGCTCTAGAGGAAGAAGCCCTG-3' and 5'-TAGAATTCAGCTCCTTGAAGAAGAT G-3', with restriction endonuclease recognition sites of *Xba *I and *EcoR *I, respectively.

β ENaC, 5'-TAATACGACTCACTATAGGGACGTTGCCATTCAGAACCTC-3' and 5'-AATTAACCCT CACTAAAGGGGCAGCCTCAGGGAGTCATAG-3' with addition of T7 and T3 RNA polymerase recognition sequence, respectively.

γ ENaC, 5'-CATCTAGACCTTCAGCTCAGGAATCAATGC-3' and 5'-CAACGAATTCTCGGAAGCCT CAGACG-3' with addition of recognition sites of *Xba *I and *EcoR *I.

The resulting PCR product of β ENaC was cloned into pUC vectors (Stratagene, Germany), which were digested by *Sma *I. The α and γ ENaC PCR fragments were digested with *Xba *I and *EcoR *I and cloned into the pBluescript II KS (-) vector (Fermentas, Germany), which provided the needed RNA polymerase recognition sites. In our experiments, gustducin mRNA served as positive control. One clone containing partial rat gustducin fragment was kindly provided by Robert F. Margolskee [21] and it was used to produce gustducin probes. After ENaC-, and gustducin-containing plasmids were linearized, cRNA probes were generated by *in vitro *transcription using digoxigenin RNA labeling mix (Roche) and T3 (antisense) or T7 (sense) RNA polymerase (Fermentas).

#### *In situ *hybridization

*In situ *hybridization was performed as that described before [45]. 10 μm-thick slices of rat tongues containing fungiform, foliate and circumvallate papillae, were sectioned and thaw-mounted onto SuperFrost plus slides (Menzel-Gläser, Germany). Prior to hybridization the sections were postfixed, permeabilized, and acetylated. Prehybridization was carried out at 55°C for 5 h, followed by hybridization of cRNA probes at 55°C overnight. After hybridization the slides were washed several times at low stringency, followed by RNAse A treatment and high stringency washes using 0.4 × SSC buffer at 55°C. Hybridized cRNA probes were detected using an anti-digoxigenin antibody and colorimetry. The signal specificities of mRNA for each gene in the taste tissues were tested by using a sense probe as a negative control. Images were generated with a CCD camera (RT slider, Diagnostic Instruments) mounted to a Zeiss Axioplan microscope (Zeiss, Oberkochen, Germany).

### Real-time quantitative RT-PCR

Real-time RT-PCR was performed by the ABI PRISM/7300 Sequence detection systems (Applied Biosystems, USA), using the TaqMan^® ^Universal PCR Master Mix No AmpErase^® ^UNG (Applied Biosystems) and specific oligonucleotide primer/probe sets, which were designed from the sequences in the GenBank database using Beacon Designer software (PREMIER Biosoft International, USA). The primers were chosen to span an intron to avoid the detection of any contamination of genomic DNA. TaqMan probes were labeled at the 5'-end with the fluorescent reporter dye, (FAM) and at the 3'-end with the quencher dye, (TAMRA). The sequences of primers and probes are shown in Table 2. All primers and probes were synthesized at MWG, Germany.

**Table 2:** Sequences of primer pairs and probes used for the real-time RT-PCR

Gene GenBank Accession #	Sequence 5'-3'	Fragment size (bp)
**α rENaC (**NM_031548**)**		96
Forward primer	GAGTCTCCTTCTGTCACGATGG	
Reverse primer	CATCTCCACCACAGAGAGCAC	
TaqMan probe	CCAAACCACAGGCTCCACTGGCTGC	
**β rENaC (**x77932**)**		99
Forward primer	CCAATGGGACCGTGTGTACC	
Reverse primer	GCACTTGTGAGAAGATGTTGGTG	
TaqMan probe	CCGAAACTTCACCAGTGCCACCCAGG	
**γ rENaC (**NM_017046**)**		106
Forward primer	AGCCAAGGTGCTTATCCATCAG	
Reverse primer	TTCTGTCAGGTGCATTCCTATGG	
TaqMan probe	CATTGCTGTCTCGATCTCCATCCCCACG	
**rBDNF (**NM_012513**)**		97
Forward primer	TCAGCAGTCAAGTGCCTTTGG	
Reverse primer	CGCCGAACCCTCATAGACATG	
TaqMan probe	CCTCCTCTGCTCTTTCTGCTGGAGGAATACAA	
**rTrkB (**M55292**)**		100
Forward primer	TTTGTGGCTTACAAGGCGTTTC	
Reverse primer	GGTGGCGGAAATGTCTCCTG	
TaqMan probe	AAGAACGGCAACCTGCGGCACATCAA	

The real-time PCR reactions were conducted using 25 μl total volume with 1 × TaqMan Master Mix (Applied Biosystems), 720 nM forward and reverse primers, 200 nM probe, and target cDNA. To determine the PCR efficiencies rat lung (containing ENaC subunits) and brain cortex (containing BDNF and TrkB) cDNA were serially (10-fold) diluted to perform PCR in parallel. For normalization of cDNA loading, all samples were run in parallel with the housekeeping gene, Glyceraldehyde-3-phosphate dehydrogenase (GAPDH) designed and synthesized by Applied Biosystems "TaqMan Gene Expression Assay" customer service. Each assay was carried out in triplicate. Amplification of cDNA was performed for 40 cycles, the first cycle was performed at 95°C for 10 min. Cycles 2–40 were performed at 95°C for 15 s, followed by 60°C for 1 min.

### Data analysis

Results are expressed as mean ± SEM. For real-time RT-PCR, The normalized expression of target gene was calculated as normalized expression = (*E*_target_)^ΔCT^_target (control – sample)_/(*E*_ref_) ΔCT_ref (control – sample)_, where *E*_target _is reaction efficiency of the gene of interest; *E*_ref_, reaction efficiency of the reference gene; and ΔCT, the cycle difference between the control and the sample. The significance of difference between groups was evaluated by ANOVA or randomization test (50000 random reallocations), which was performed with REST [46]. A *p *value of less than 0.05 was considered to be statistically significant. All experiments were performed at least twice to get the same results.

## Authors' contributions

TH conceived the study, collected animal samples, carried out all *in situ *hybridization and real-time RT-PCR experiments, data analyses and drafted the manuscript. FS participated in the study design, the animal sample collection, data analyses and manuscript polishes. All authors read and approved the final manuscript.
